# The intersection of mitochondrial dynamics and sarcopenic phenotyping: A call for mechanistic detail

**DOI:** 10.1016/j.tjfa.2025.100116

**Published:** 2025-11-26

**Authors:** Parth Aphale, Himanshu Shekhar, Shashank Dokania

**Affiliations:** Dr. D.Y. Patil Vidyapeeth (Deemed to be University), Pimpri, Pune, Maharashtra, India

We read with great interest the article by Springer-Sapp et al. on the impact of sarcopenia and 12 weeks of resistance exercise training (RT) on skeletal muscle mitochondrial quality control proteins [1]. The authors provide valuable, well-executed data suggesting that mild-to-moderate sarcopenia, as defined by the appendicular lean mass to body mass index (ALM/BMI) ratio, does not substantially alter the baseline expression of mitochondrial fusion, fission, or mitophagy markers compared to non-sarcopenic controls. Furthermore, the observed strength gains (13 %) following RT were not universally coupled with robust changes in these proteins, aside from a trend toward increased mitofusin 2 (Mfn2). However, this work invites further critical discussion regarding methodological nuances that may influence the interpretation of mitochondrial adaptation in aging muscle.

First, the inverse correlation noted between muscle mass metrics and the expression of Complex IV (CIV) and the fusion protein Opal-S is intriguing. While the authors propose compensatory fusion or disrupted membrane potential, the finding warrants closer functional investigation. It raises the question of whether this increased mitochondrial content in the remaining, smaller fibers represents a localized mitochondrial crowding phenomenon due to selective atrophy of type II fibers, or if it reflects an enhanced energy demand per unit of remaining muscle tissue [2,3]. Future work should integrate single-fiber analysis of mitochondrial respiration and quality to resolve this counter-intuitive correlation.

Second, the definition of sarcopenia by ALM/BMI alone merits scrutiny, especially given that strength increased significantly without concomitant hypertrophy. The disconnect between neural-driven strength gain and the lack of mitochondrial or mass adaptation suggests the participants were at a stage of *dynapenia* rather than severe sarcopenia. As current clinical guidelines increasingly prioritize muscle *strength* and *function* over mass alone for adverse outcome prediction, relying on an index of muscle quantity, particularly one that did not correlate with functional indices like VO2max​ in the overall cohort, limits the clinical translational power of the mitochondrial findings [4]. Would the mitochondrial proteomic signature differ if the groups were categorized strictly by low strength, independent of mass?

Finally, the timing of the post-RT muscle biopsy 24 to 48 h after the last bout is critical. The lack of change in chronic adaptation markers like PGC-1$\alpha$ and most mitochondrial dynamics proteins aligns with studies suggesting that the *chronic* physiological signal for mitochondrial biogenesis typically manifests later or requires a specific recovery period distinct from the post-exercise acute signaling phase [5. Given that a single bout of RT can acutely perturb these proteins, the measurement window may capture a transient state of protein remodeling rather than the final adaptive equilibrium achieved over 12 weeks.

In conclusion, the study provides an important null hypothesis regarding the simplicity of mitochondrial quality control disruption in mild sarcopenia. To advance this field, we suggest future designs incorporate gold-standard measures of mitochondrial ultrastructure (e.g., cristae density and connectivity via sophisticated TEM analysis) beyond simple area quantification, alongside functional sarcopenia criteria, to better link molecular expression to clinical outcomes.

## Funding and disclosures

None.

## Declaration of interest

None.

## Declaration of generative AI

The authors declare that AI-based language assistance was used solely for grammar refinement. All intellectual content, interpretation, and conclusions are the sole responsibility of the authors.

## CRediT authorship contribution statement

**Parth Aphale:** Conceptualization, Supervision, Writing – original draft, Writing – review & editing. **Himanshu Shekhar:** Visualization, Writing – original draft, Writing – review & editing. **Shashank Dokania:** Writing – review & editing.

## Declaration of competing interest

The authors declare that they have no known competing financial interests or personal relationships that could have appeared to influence the work reported in this paper.
